# Hygrothermal and Acoustical Performance of Starch-Beet Pulp Composites for Building Thermal Insulation

**DOI:** 10.3390/ma11091622

**Published:** 2018-09-05

**Authors:** Hamzé Karaky, Chadi Maalouf, Christophe Bliard, Tala Moussa, Nadim El Wakil, Mohammed Lachi, Guillaume Polidori

**Affiliations:** 1 Groupe de Recherche en Sciences de l’Ingénieur GRESPI, SFR Condorcet FR CNRS 3417, Université de Reims Champagne Ardennes, Moulin de la Housse, 51687 Reims, France; chadi.maalouf@univ-reims.fr (C.M.); tala.moussa@univ-reims.fr (T.M.); nadim.el-wakil@univ-reims.fr (N.E.W.); mohammed.lachi@univ-reims.fr (M.L.); guillaume.polidori@univ-reims.fr (G.P.); 2 Institut de Chimie Moléculaire de Reims, ICMR-UMR 7312 CNRS, Université de Reims Champagne Ardennes, Moulin de la Housse, 51687 Reims, France; christophe.bliard@univ-reims.fr

**Keywords:** bio-based composite, starch–beet pulp, porosity, thermal conductivity, hygrothermal performance, acoustical performance

## Abstract

This article deals with the elaboration and the characterization of an innovative 100% plant-based green composite made solely of beet pulp (BP) and potato starch (S). Using this type of material in insulation applications seems a good solution to reduce the CO_2_ gas emissions in building. The influence of the starch amount on composite characteristics was studied. Four mixtures were considered with different S/BP mass ratios (0.1, 0.2, 0.3 and 0.4). The physical properties of these materials were studied in terms of porosity, apparent and absolute densities, thermal conductivity, and hygric properties. The influence of humidity content on acoustical properties was studied as a function of frequency. Test results show a real impact of both starch and humidity contents on the hygrothermal and acoustical properties of the studied material due to the porosity. The composite with the lowest amount of starch (S/BP = 0.1) seems to be the optimal composition in terms of the hygrothermal and acoustical behaviors.

## 1. Introduction

The building sector is responsible for approximately 50% of the total energy consumption and 11% of CO_2_ gas emissions. Therefore, it has become crucial to turn to renewable energy and resources as well as eco-friendly and sustainable materials especially in building applications in order to reduce both environmental impacts and primary energy use. The recent use of bio-based composites in construction appears to be an innovative solution while maintaining high indoor comfort [1,2,3,4,5,6].

Several studies have been already published on the use of crop by-products for buildings such as wheat straw, which was used in sustainable building [7]. Hemp concrete is the most studied among different composite materials [8,9,10,11,12]. Hemp–starch composite presents a porous structure providing a good thermal insulation with a thermal conductivity ranging from 0.06 W·m^−1^·K^−1^ to 0.1 W·m^−1^·K^−1^. It is also classified as an excellent humidity regulator. The moisture buffering value (MBV) measured ranges from 2.6 g·m^−2^ % RH^−1^ to 2.7 g·m^−2^·% RH^−1^. The impacts of both particle size and starch content were also studied [8]. The water vapor permeability of the hemp-lime concrete obtained by Collet et al. [4] is quite high (1.7 × 10^−11^ kg·m^−1^·s^−1^·Pa^−1^).

The porosity of several bio-based composites as well as the influence of both porosity and water content on the composite properties was investigated [2,8,13]. The results show that the hygrothermal properties of composite increase with its porosity. In another study, the hygric properties such as sorption isotherm, water vapor permeability, and MBV of bio-based materials (for example, hemp concrete, flax concrete and rape straw concrete) were investigated [14]. The results showed that these materials have a low thermal conductivity but a high MBV, which provides a good hygrothermal insulating capacity.

Many studies have investigated the acoustical performance of hemp–starch composites [11,15,16,17]. The hemp–starch composite is a good acoustical insulator due to its porous structure; it can absorb 70% of the sound waves for medium and high frequencies. The sound absorption coefficient is independent of the composite porosity. In this paper, the authors deal with the study of starch–beet pulp (S-BP) composite for building applications.

**Figure 1:**
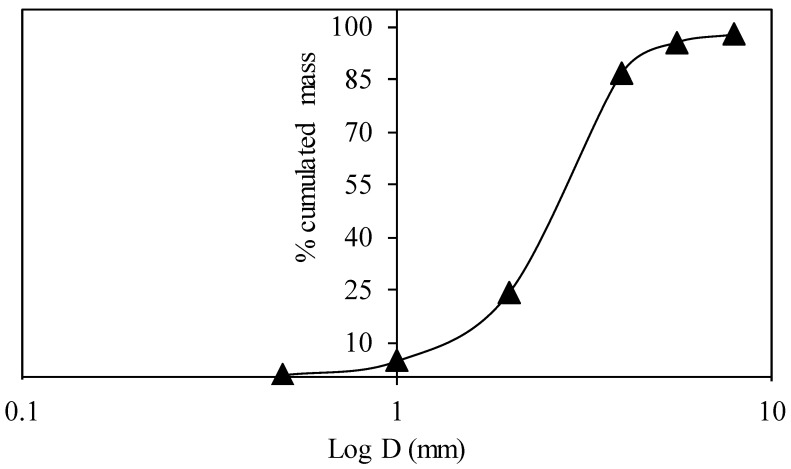
Grain size distribution curve of BP.

Sugar beet (*Beta vulgaris*) is widely grown in France (33.8 million tons in 2016) and used in sugar factory to produce the white sugar [18,19]. Sugar beet pulp is a by-product and mainly used to feed livestock due to the high nutritional value [18,19]. It is mechanically compressed using an extrusion machine to obtain the extruded beet pulp (BP), which has been used in this article to produce the S-BP composite. The dominant size of the dried extruded beet pulp lies between 2 mm and 4 mm (Figure 1) and it presents a rough surface, which provides a good adhesion with starch binder. BP is lightweight and a porous material (Table 1) (Absolute density, *ρ_abs_* = 1073.4 kg·m^−3^), it is lighter than hemp shiv (*ρ_abs_* = 1443 kg·m^−3^) [20]. The porosity of BP was measured using a cyclohexane method (paragraph 1.4). BP contains mainly pectin, hemicellulose, and cellulose and it is an important source of pectin, which has been evaluated in many studies [21,22,23]. It is used in food, cosmetics industries, and as a bio-adsorbent for the removal of heavy metals [24]. Monreal et al. [25] studied cement-beet pulp concrete, where an important dimensional deformation was observed. Many physico-chemical treatments were carried out for the beet pulp to reduce its hydrophilic nature. The linseed oil treatment was the optimal treatment, which reduces the ability of aggregates to absorb water and swell.

Starch is a polysaccharide that is converted to sugar as a result of hydrolysis and contains two polymers with different primary structure, Amylose (linear chain) where glucose units are joined by *α* 1–4 glycosidic bonds and amylopectin (branched chain) where the glucose chains are branched onto α 1–6 positions [26]. Starch is a hydrophilic material and exists in many plants such as cereals (30% to 70%), tubers (60% to 90%) and legumes (25% to 50%) [27,28,29]. It is used in paper factory, textile and food factories via the beverage, and confectionery and baked goods industries [30,31]. It can be also used to produce bio-ethanol and pharmaceutical products by fermentation process [32]. The present work aims to elaborate a bio-sourced material made of BP and potato starch designed to be used in the building sector for walls and ceiling insulation applications. The hygrothermal and acoustical characteristics of this material are determined and fitted analytically.

## 2. Materials and Methods

### 2.1. Extruded Beet Pulp (BP) 

**Figure 2:**
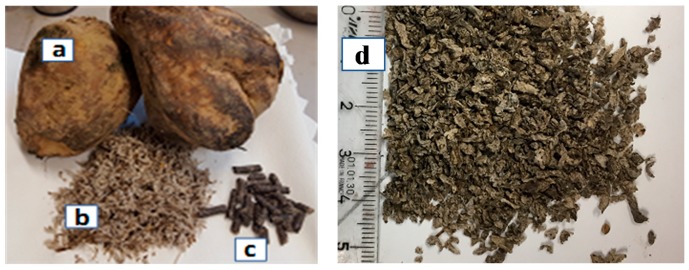
(**a**) Sugar beet fruit; (**b**) fresh sugar beet pulp; (**c**) extruded beet pulp pellets and (**d**) dried extruded beet pulp.

**Figure 3:**
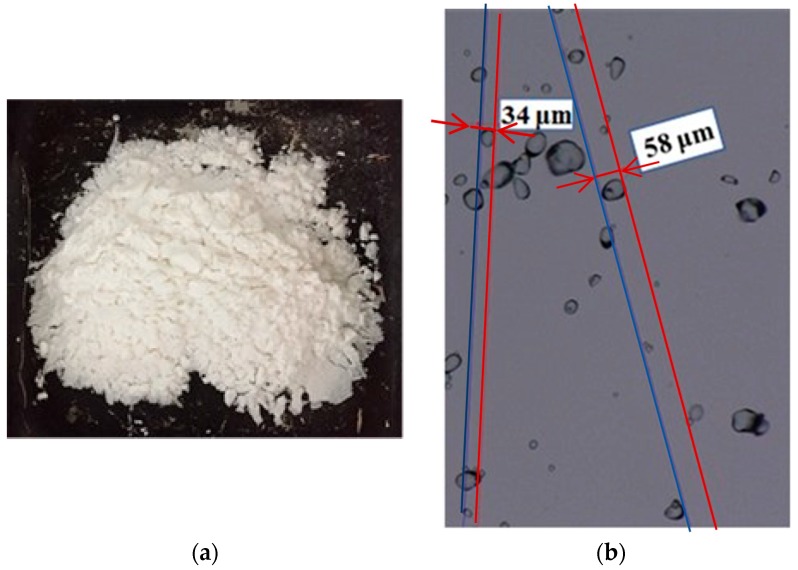
(**a**) Powder of potato starch; (**b**) potato starch grains seen by optical microscopy.

The 8–10 mm diameter extruded BP pellets (18% humidity) (Figure 2) were provided by Cristal Union factory (Pomacle, France). To ensure proper conservation in the laboratory, the pulps were kept at −20 °C until use. Before use, the extruded BP was immersed in water for two hours and then dried at 50 °C for two days (Figure 3).

### 2.2. Potato Starch

Potato starch was purchased from Roquette, Lestrem, France. Potato starch (Figure 3a) has a high polymerization degree, which gives a viscous binder and provides a good mechanical property for the S-BP composite. Starch was used as a binder to make several bio-composites, such as hemp–starch and palm date fibers-starch [8,10,11,33]. It can stick the particles together and ensure the transmission of shear forces between the fibers. The starch grains can permeate the BP pores because of their smaller size (Figure 3b) and thus provide a good adhesion between the components.

### 2.3. Composite Formulations

Starch and sugar beet pulps are hydrophilic materials, due to their great ability to store water molecules in their structures. This imposes a competition between these two components to absorb water from the mixture resulting in a binder with undissolved starch grains and a mixture of wet pulps. Several studies have proposed to prepare the binder separately, with optimum dynamic viscosity and surface tension [11,17]. In the present study, this solution was not effective enough, due to the hydrophilic behavior of the pulps that causes a significant water gradient all around the sample during drying, and consequently results in dimensional deformations. For this reason, a new preparation method was used that does not require any water addition. To avoid the water competition between beet pulps and starch grains, the extruded pulps were soaked in distilled water to ensure saturation with a water/BP mass ratio of 2.5 [25]. The wet BP is then mixed with starch powder. The mixture was kept in an autoclave to dissolve the starch under water vapor pressure. After that, the samples were compacted using the traction machine INSTRON 8801 (INSTRON, Norwood, MA, USA) at 44 kPa. Finally, the mixture was frozen and dried using a freeze dryer (Alpha 1/2-4 CHRIST, GROSSERON, Coueron, France), then put in a climatic chamber at 50 °C and 10% RH to continue the drying procedure.

**Figure 4:**
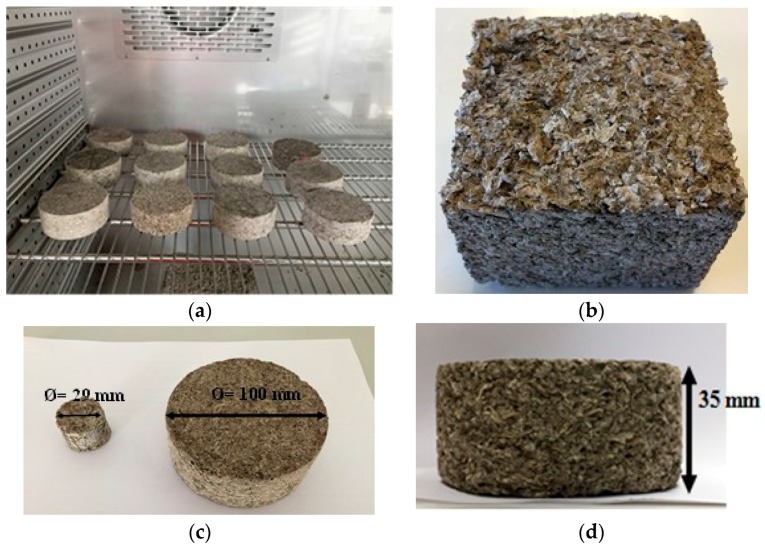
Composite specimens obtained from the four formulations. (**a**) Cylindrical samples; (**b**) Cubical samples; (**c**,**d**) Samples for acoustical test.

Four formulations with four different S/BP mass ratios (0.1, 0.2, 0.3 and 0.4) were prepared to study the influence of starch amount on the composite behavior (Figure 4).

### 2.4. Density and Porosity

The absolute density of S-BP composite was determined using the pycnometer method [2,8]. The pycnometer was filled with a given mass of dried pulp and half of its volume with cyclohexane, which is a non-polar solvent and does not affect the composition and mass of the pulp. The system underwent six cycles of boiling (30 min) and cooling (10 min); during these cycles, air escaped from the pulp leaving its pores, and cyclohexane occupied the pore spaces; and during the sixth cycle, the system was kept under an argon atmosphere to avoid humidity. At room temperature (20 °C), the pycnometer was filled to the end and plugged with the stopper. The system was then weighed with an accuracy of 10^−3^ g. The absolute density was calculated using Equation (1):

(1) $$\rho_{abs}=\frac{M_{1}\times \rho_{cyc}}{M_{1}-\left(M_{2}-M_{3}\right)}$$

where *ρ_abs_* is the absolute density (kg·m^−3^), *ρ_cyc_* is the density of cyclohexane (kg·m^−3^), *M*_1_ is the dry mass of aggregates, *M*_2_ is mass of the pycnometer filled with cyclohexane and saturated aggregates, and *M*_3_ is the mass of pycnometer and cyclohexane.

Precautions were taken to avoid the residual moisture accumulation in the reflux system. Each measurement was performed at least three times to be considered representative.

The porosity and pore structure of the composite was measured using mercury intrusion porosimetry (MIP) using a 140 series Pascal Thermo Scientific Porosimeter (France Scientifique, Saint Genis Laval, France) [10,34]. The pore access diameter ranged from 3.8 μm to 1000 μm.

### 2.5. Sound Absorption Coefficient

**Figure 5:**
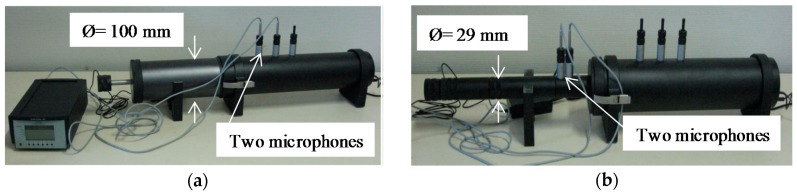
Kundt tube used to measure the sound absorption coefficient for (**a**) low frequencies and (**b**) high frequencies.

The Kundt tube type BK 4206 (BK 4206, BKSV, Nærum, Denmark) (tube with two fixed microphones) was used to measure the sound absorption coefficient *α*. This device consists of a cylindrical tube with two quarter inch BK type 4187 microphones, a BK 2706 power amplifier and an OROS analyzer (38, Oros, Meylan, France). For the sound absorption measurements, two tubes of varied sizes were used to change the frequency ranges (Figure 5).

The Kundt tube is able to measure the acoustic absorption as well as the surface impedance according to the NF EN ISO 10534-2 standard [35]. The device used for this test contains two microphones spaced a variable distance depending on the tube, a loudspeaker attached to an extremity of the tube, and the sample placed on the other extremity. By using the analyzer generator (38, OROS, Meylan, France) and power amplifier (BK 2706, BKSV, Nærum, Denmark), the speaker was excited with white noise. Both microphones detect reflected and incident sound pressures. The principle of the measurement of sound absorption is therefore based on the study of the transfer function between two signals picked up by two microphones.

### 2.6. Thermal Conductivity

**Figure 6:**
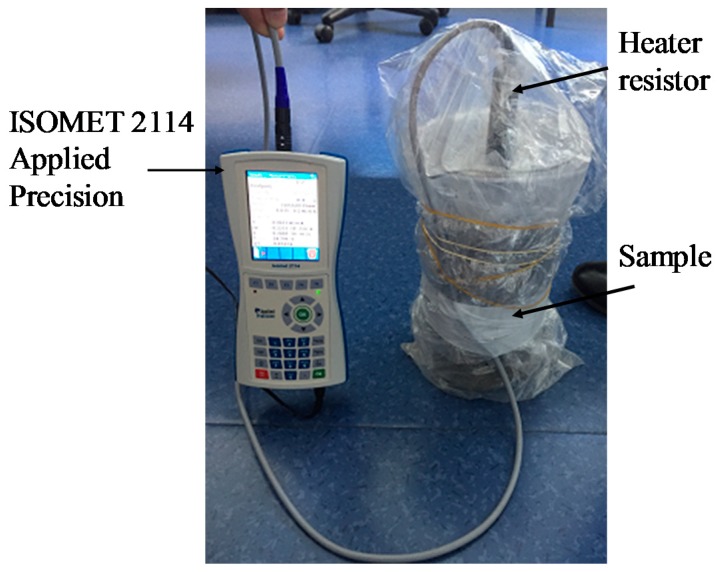
Thermal conductivity measurement of S-BP composite.

The thermal conductivity of S-BP composite was measured using ISOMET 2114 Applied Precision (Isomet 2114, Aventech, Buc, France) [36]. The heat flow was generated by heating an electrical resistor inserted into the sample hole to ensure a direct heat contact with the sample. The thermal conductivity evaluation is based on the temperature measurements taken periodically as a function of time. The tested cubical samples (Figure 4) were dried using a climatic chamber (MKF 720 Binder, Labo and Co, Marolles En Brie, France) at 50 °C and 10% RH. Before the measurement, the dried samples were cooled and stabilized at 23 °C and 10% RH. The samples were covered during the test to avoid the humidity absorption (Figure 6).

**Figure 7:**
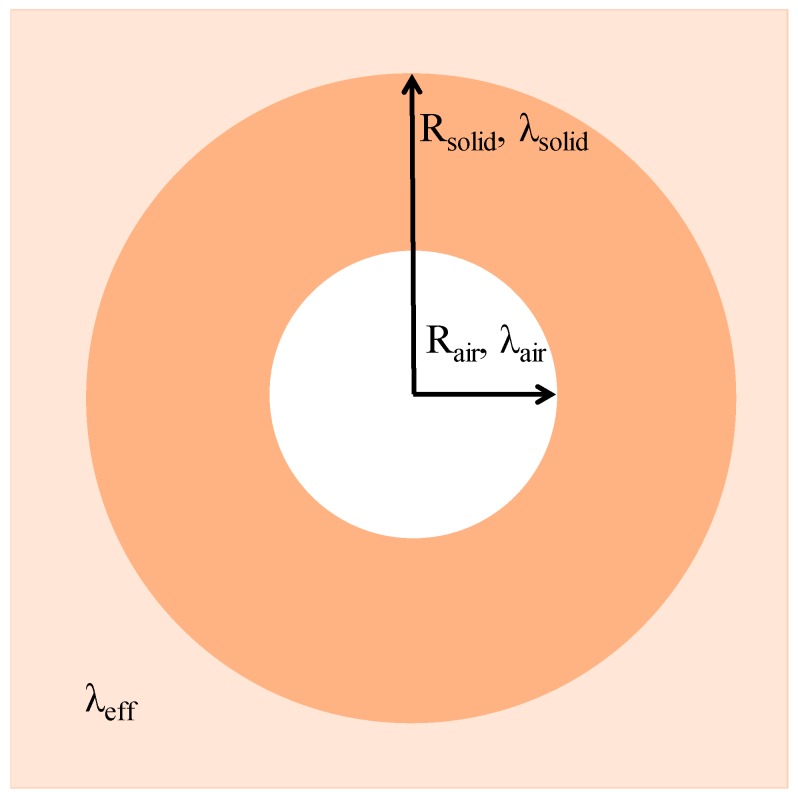
Agro-material geometry considered as a two-phase medium in a homogeneous equivalent medium.

Regarding the theoretical thermal conductivity for the agro-materials solid phase, it was evaluated at 20 °C using the representation of Collet [37] and Rahim [14] shown in Figure 7. The effective thermal conductivity, *λ_eff_* was determined as a function of the thermal conductivities of the solid phase and the air, using Equation (2), where *n* is the total porosity, *λ_a_* is the air thermal conductivity, and *λ_s_* is the solid phase thermal conductivity.

(2) $$\lambda_{eff}=\lambda_{s}\times \left(1+\frac{n}{\left(\frac{1-n}{3}\right)+\frac{1}{\left(\lambda_{a}/\lambda_{s}-1\right)}}\right)$$

The heat capacity of the S-BP composite was measured using a C80 Calvet calorimeter from Setaram Instrumentation (FRANCE). In the calorimetric detector, the sample and the cell reference were completely surrounded by an array of thermocouple detectors for the heat transfer measurements including radiation, convection, and conduction.

### 2.7. Permeability

**Figure 8:**
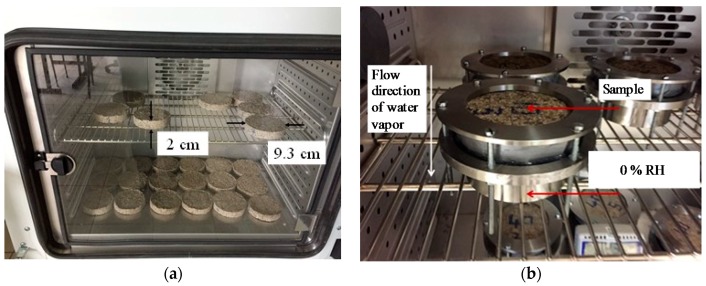
(**a**) Samples in climatic chamber at 50 °C and 10% RH; (**b**) dry cup method for vapor permeability measurements.

Water vapor permeability *δ_v_* (kg·m^−1^·s^−1^·Pa^−1^) represents the capacity of water vapor to pass through the material under steam flow pressure. The measurement was carried out according to the NF EN ISO 12571, using the dry cup method [2,11]. The samples were dried in a climatic chamber at 50 °C and 10% RH (Figure 8a). The sample was sealed at the top of the cup, which contained the silica gel at its bottom, providing a 0% RH (Figure 8b). The sample assembly was placed in a climatic chamber set to 50% RH at 23 °C. The water vapor resistance factor *μ* and *δ_v_* are respectively given in Equations (3) and (4), where *G* is the mass rate (kg·s^−^^1^), Δ*P_v_* is the vapor pressure gradient, *e* is the thickness of the sample, *A* is the exposed surface area (m^2^), and *δ_a_* is the air water vapor permeability.

(3) $$\delta_{v}=\frac{G\times e}{\Delta P_{v}\times A}$$

(4) $$\mu =\frac{\delta_{a}}{\delta_{v}}$$

### 2.8. Sorption Isotherm

The sorption isotherm tests were carried out in accordance with NF EN ISO standard 12572 (2001) [2,10]. They allow plot the sorption curve representing the variation of the water content as a function of relative humidity of ambient air at a constant temperature of 23 °C. The sorption isotherm shows, in other terms, the equilibrium between water content in the composite and relative humidity [38].

Four cylindrical samples of 10 cm diameter and 4 cm thickness per mixture were prepared (Figure 4). They were dried for seven days at 50 °C and 10% RH until they reached the dry state. Then they were placed in a climatic chamber at 23 °C and at various RH levels: 20, 40, 60, 80 and 92%. At each humidity level, the measurements were carried out until the variation for three successive mass readings became less than 0.1% of the total mass. The experimental results were then fitted with three analytical models: GAB (Guggenheim-Anderson-de Boer) [39], Merakeb [40] and Van Genuchten [41].

Experimental data were correlated with the least squares method. To estimate the variability attributed to each model, the correlation coefficient (*R*^2^) was calculated. The mean deviation *E* and root mean square error (*RMSE*) were also evaluated. These criteria allowed judge the quality of the adjustment of experimental results with respect to the model. They are respectively defined in Equations (5) and (6).

(5) $$E=\frac{100}{N}\times \Sigma_{i=1}^{n}\frac{\left|m_{e}^{i}-m_{p}^{i}\right|}{m_{e}^{i}}$$

(6) $$RMSE=\sqrt{\frac{\Sigma_{i=1}^{n}{\left(m_{e}^{i}-m_{p}^{i}\right)}^{2}}{N}}$$

where *m_e_* is the experimental measure, *m_p_* is the value computed using the model and *N* is the total number of experimental values. The adjustment between experimental and analytical values is considered correct when the mean relative deviation does not exceed 10%. The adjustment quality is inversely proportional to *E* and *RMSE* values.

### 2.9. Moisture Buffer Value (MBV)

*MBV* represents the capacity of the composite to regulate the relative humidity of a medium. The Nordtest protocol defines cyclic step-changes in relative humidity after stabilization, between high (75%) and low (33%) values for 8 h and 16 h, respectively [8,9,10,12,42].

Four 10 cm diameter and 4 cm thick cylindrical samples were tested for each formula. The edges and back-sides of the samples were sealed with duct tape to obtain a one-dimensional moisture flow. The samples were stabilized at 23 °C and 50% RH in a climatic chamber and weighed until they reached equilibrium. During the periodic exposure, the samples were weighed five times during adsorption phase and twice during desorption phase. When the mass variation between three consecutive days became below 5%, the experiment was stopped and the *MBV* was calculated using Equation (7):

(7) $$MBV=\frac{\Delta m}{A\times \left(RH_{high}-RH_{low}\right)}$$

with *A* (m^2^) is the sample area that is in contact with air. *RH_high_* and *RH_low_* respectively represents high relative humidity (75% RH) and low relative humidity (33% RH), and Δ*m* represents the mass change during the adsorption/desorption phase (g).

## 3. Results and Discussions

### 3.1. Porosity Analysis

**Figure 9:**
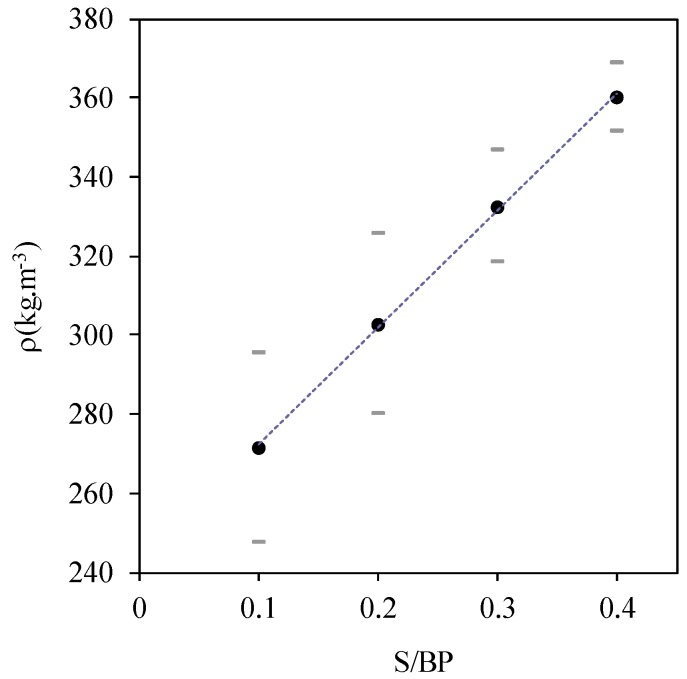
Apparent density evolution with S/BP mass ratio.

Figure 9 shows the apparent density variation of the fully dried composite material in the climatic chamber at 50 °C and 10% RH, as a function of the S/BP mass ratio. It can be observed that the density increases linearly with the S/BP mass ratio. The apparent density varies from 271.4 kg·m^−3^ to 360 kg·m^−3^. It should be noted that the average apparent density of beet pulp–cement concrete is between 570 and 770 kg·m^−3^ [25]. The apparent density (ρ) of the samples can be expressed as a function of the S/BP mass ratio between 0.1 and 0.4 by Equation (8).

(8) $$\rho =295.42\times \left(\frac{S}{BP}\right)+242.8$$

**Figure 10:**
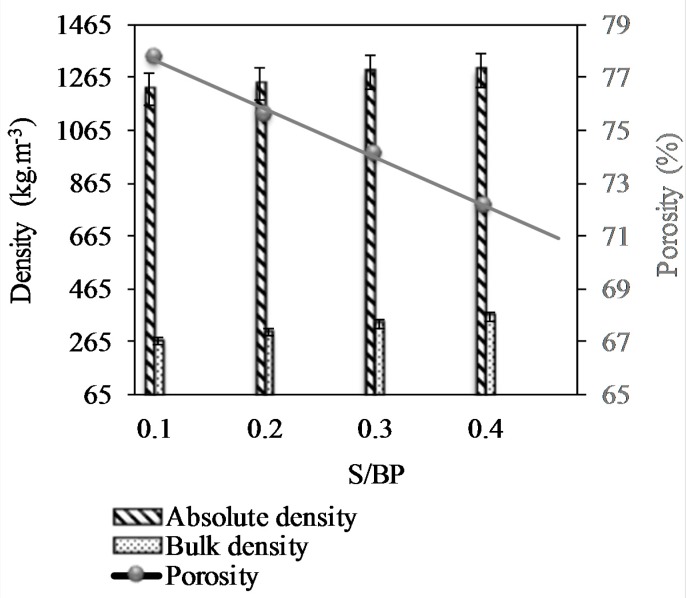
Density and porosity of S-BP composite measured by cyclohexane method.

The density and porosity results are shown in Figure 10. The absolute and apparent densities increased with the S/BP ratio. However, the porosity of the composite decreased logarithmically. Therefore, the sample with the lowest amount of starch (S/PB = 0.1) has the highest porosity (79.75%) and the lowest absolute density (*ρ_abs_* = 1222 kg·m^−3^). The total porosity was between 70.60% and 79.75% and the decrease in the S/BP ratio increased the total porosity. The hemp–starch composite shows a higher porosity than the beet–starch composite. It can be explained by the fact that the hemp shiv size is greater than that of the beet pulp [8]. The influence of starch is in agreement with the results obtained by Rahim et al. [14] and Bourdot et al. [8]. The presence of starch aerogel increases the apparent density and decreases the total porosity by filling the inter-particle space between the pulp particles sealing the pores. Therefore, the composite would contain closed pores as well as more or less accessible open pores.

Bourdot et al. studied the density and porosity of hemp particles of two different sizes 0–5 mm and 0–20 mm, the results showed that the small particles have a density (1266 kg·m^−3^) and a porosity (89.3%) smaller than those (1271 kg·m^−3^ and 91.3%, respectively) of the large particles [8]. The absolute density and porosity of the hemp–starch agro-materials were similar (approximately 1240 kg·m^−3^ and 89%, respectively) whatever the composition. However, the absolute density increases slightly with the hemp/starch ratio (H/S) and the proportion of 0–20 mm hemp [4,10]. The presence of potato starch gel increases the apparent density and decreases the total porosity by filling the pore spaces between aggregates. This can be explained by the creation of closed pores between the aggregates and the gel. It can thus be noted that the agro-materials are composed of closed and more or less accessible open pores. The MIP (mercury intrusion porosimetry) method was used to determine the accessible porosity, which influences the agro-material properties, in particular the hygroscopic properties.

**Figure 11:**
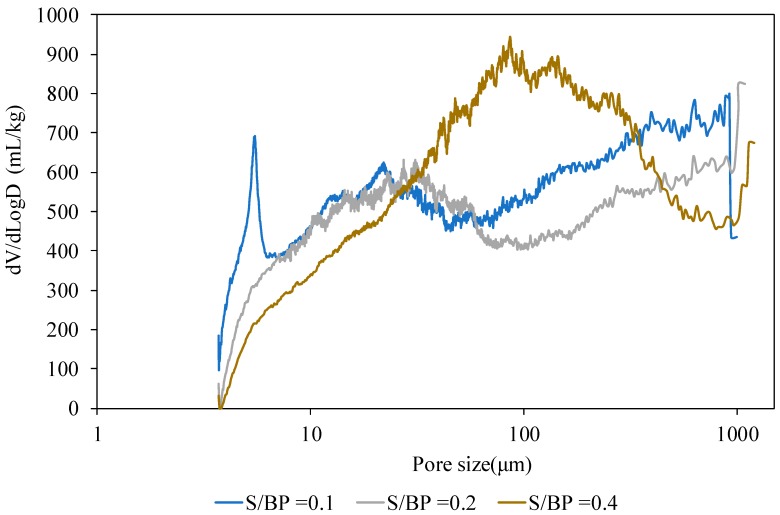
Pore size distribution in S-BP composites with different S/BP ratios.

Mercury porosimetry allowed us to study the influence of the S/BP mass ratio. The mercury first filled the small pores and then the large pores. Figure 11 shows the analysis of the measurement of porosity under mercury pressure. It should be noted that the accessible porosity of the starch-BP material is bonded to the binder due to the porous nature of the starch gel as deduced from the results obtained with the pycnometer method. The sample with a mass ratio of S/BP = 0.1 was the most porous with a total porosity of 66%. The porosity of the composite decreased with the increase in the S/BP mass ratio to reach a value around 60%. The pore distribution of each composition shows that the volume of the pores (350–1000 μm) decreased with the increase in the binder content, which can be explained that the starch binder fills the pores and reduces their volume. Thus, the existence of the macro-pores in the range (350–1000 μm) is entirely due to the morphology of the aggregate and the starch content. The volume of the pores in the range of diameters lower than 350 μm increased with the starch amount. In fact, during the drying step, water escapes from the sample and creates capillary voids between the matrix and the starch—the binder continues to plug these composite voids. The porosity measured by the pycnometer method decreased linearly from 77.79% to 72.16% and the absolute density increased from 1222.18 kg·m^−3^ to 1293.42 kg·m^−3^ when the S/BP mass ratio increased from 0.1 to 0.4. The absolute mass of the potato starch which is around 1451.19 kg·m^−3^ explains the increase in the absolute mass of the composite.

### 3.2. Sound Absorption Coefficient

The sound absorption coefficient of the bio-composite depends on the pore distribution, the connectivity between the various porous networks, and the type of the binder [15,33].

**Figure 12:**
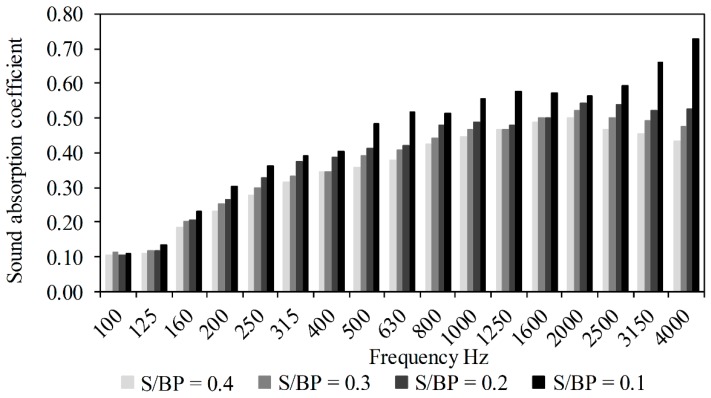
Sound absorption coefficient as a function of frequency.

In the present study, four formulations with different S/BP mass ratios were analyzed to investigate the influence of starch amount and humidity content. Figure 12 shows the results obtained using Kundt tube. For medium and high frequencies, the sound absorption coefficient decreased when the S/BP mass ratio increased from 0.1 to 0.4. It suggests that the starch binder fills the pores and decreases the composite ability to dissipate the sound waves.

The highest sound absorption coefficient recorded for the S-BP composite achieved was about 0.72 at 4000 Hz with the S/BP mass ratio equal to 0.1. For the medium frequencies, the sound absorption coefficient of the composite was 0.6. At the same frequency, the sound absorption coefficient of the hemp–starch and cork composites was found as 0.4 and 0.28, respectively [13,15,33].

**Figure 13:**
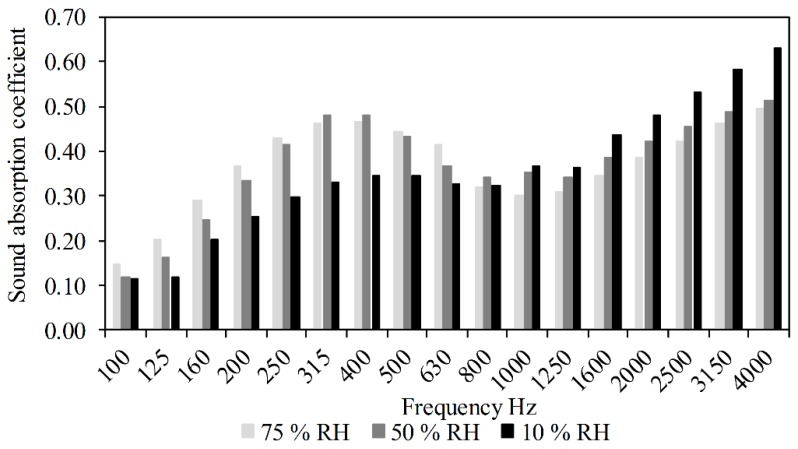
Sound absorption coefficient of S-BP composite as a function of relative humidity.

The apparent histogram in Figure 13 represents the variation of the acoustic absorption coefficient *α* for a composite having a S/BP mass ratio of 0.3, as a function of different relative humidity of the climatic chamber (10, 50 and 75% RH) in which the composite was stabilized at a constant temperature of 23 °C. It is noticeable that the sound absorption coefficient decreased for the frequency range of 1000–4000 Hz when the relative humidity increased from 10% to 75%. This is because the moisture content in the composite increases due to the increased relative humidity in the climatic chamber; the water molecules clog the micropores, resulting in a reduced porosity, thus reducing the ability of the composite to absorb or dampen the incident sound signals with small wavelength (high frequencies). For medium frequencies, the composite subjected to 50% RH showed a better acoustic performance, which means that the S-BP pulp composite works better at ambient conditions than in other conditions. At low frequencies, the acoustic behavior of the composite was reversed, the sound absorption coefficient increased with the relative humidity. This suggests that at a high humidity (75% RH) the binder existing in the macro pores is plasticized and able to dampen sound waves with long wavelength (low frequencies).

### 3.3. Thermal Conductivity, Diffusivity and Effusivity

The thermal properties of S-BP composites for different S/BP mass ratios are shown in Table 2. The results show that the thermal conductivity *λ* increased linearly from 0.069 W·m^−1^·K^−1^ to 0.075 W·m^−1^·K^−1^ when the S/BP mass ratio increased from 0.1 to 0.4. The increase of the starch amount in S-BP composite decreases the composite porosity by filling the micropores, therefore the thermal conductivity increases. The thermal conductivity *λ* can be expressed as a function of the S/BP mass ratio according to Equation (9).

(9) $$\lambda =0.0197\times \frac{S}{BP}+0.0673$$

The thermal diffusivity *a* is a physical quantity that characterizes the ability of a continuous material to transmit a temperature signal from one point to another point of the material. It is calculated using Equation (10), where *ρ_app_* is the apparent density (kg·m^−3^) and *C_p_* is the heat capacity (J·K^−1^·kg^−1^).

(10) $$a=\frac{\lambda }{\rho_{app}\times C_{p}}$$

The thermal effusivity (J·K^−1^·m^−2^·s^−1/2^) *b* represents the ability of the composite to exchange thermal energy with its environment. It is given by Equation (11):

(11) $$b=\sqrt{\lambda \times \rho_{app}\times C_{p}}$$

The thermal effusivity and diffusivity are strongly related to the binder content (starch). Increasing the binder content in the composite promotes the thermal effusivity and decreases the thermal diffusivity. Thus, the starch content increases the composite ability to store heat.

The thermal conductivity of S-BP composite is comparable to that of hemp–starch composite (between 0.063 W·m^−1^·K^−1^ and 0.100 W·m^−1^·K^−1^) [2,8]. However, it is greater than that of the other composites such as hemp–clay and cork concrete [9,12]. Thus, S-BP composite leads to a better thermal inertia compared to hemp-starch composite [2,8,9].

The self-consistent scheme was applied to estimate the beet pulp and solid thermal conductivities *λs* in agro-composites with a two phase model from the thermal conductivities measured by the Isomet method and the absolute densities and total porosities measured by the cyclohexane method. The results are shown in Table 3. The solid thermal conductivities of the composites varied between 0.26 W·m^−1^·K^−1^ and 0.30 W·m^−1^·K^−1^ whereas that of the beet pulp was 0.3151 W·m^−1^·K^−1^ which is greater than that of the starch gel (*λ_s_* = 0.2314 W·m^−1^·K^−1^). Therefore, the increase of the composite thermal conductivity is due to the increase in the starch amount and the decrease of the composite porosity results from the filling of the macro pores.

### 3.4. Permeability 

The water vapor permeability results are shown in Table 4. The increase in binder amount decreased the water vapor permeability of the S-BP composite. The permeability of the composites depends mainly on the porosity [2]. The sample with the S/BP mass ratio of 0.1 contains higher macro-pores than the other samples. Therefore, it had the higher water vapor permeability than the others. Moreover, the water vapor permeability of S-BP composites (8.90 × 10^−12^ kg·m^−1^·s^−1^·Pa^−1^) is lower than that of hemp–starch composites (1.7–6.2 × 10^−11^ kg·m^−1^·s^−1^·Pa^−1^) [4] and Typha–clay composites (2.83–6.15 × 10^−11^ kg·m^−1^·s^−1^·Pa^−1^) [2]. This is credited to the small size of the beet pulp (2–4 mm), which provides a composite more homogenous and less porous than the others.

### 3.5. Sorption Isotherm

**Figure 14:**
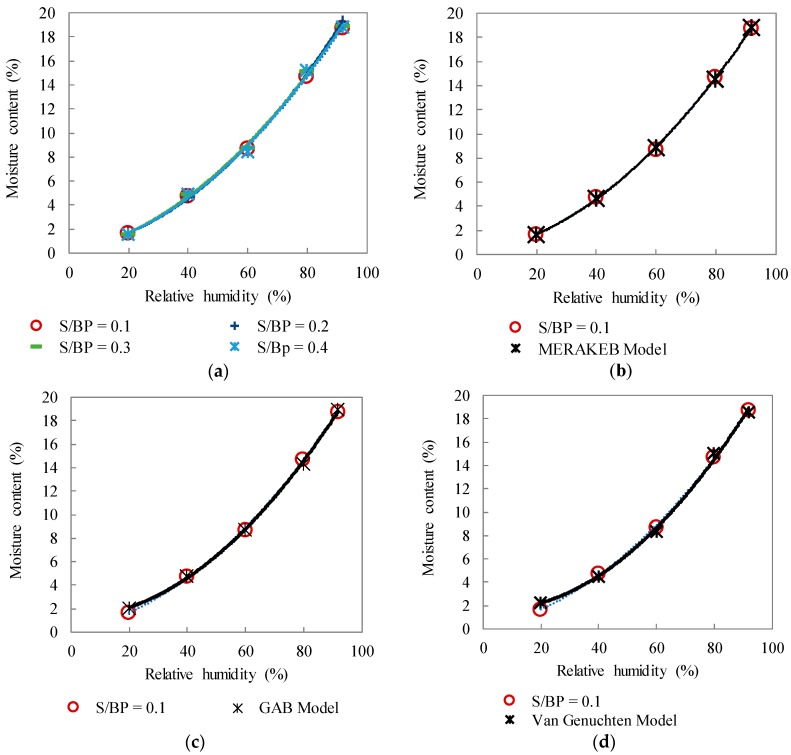
(**a**) Sorption isotherms of S–BP composites for the four formulations and the comparison of the experimental data with Merakeb (**b**), GAB (**c**), and Van Genuchten (**d**) models.

Figure 14a shows the sorption isotherm curves of different samples of the four studied formulations. These curves describe the equilibrium between the relative humidity and the humidity content of the samples at 23 °C and have a typical curve shape of cellulosic materials [43,44]. The samples show the same behavior independently of the S/BP mass ratio. This is attributed to the water sorption which depends on two parameters: the porosity and the starch amount. Increasing the S/BP mass ratio means that the starch amount increases, and the porosity decreases. These parameters balance the water sorption of the composites especially that starch is a hydrophilic material. The sorption results are comparable with those of Bourdot et al. [8].

Figure 14b–d shows the experimental values fitting according to GAB [39], Merakeb [40] and Van Genuchten [41] models. Among these models, the Van Genuchten seems to be the least close to the experimental values.

Table 5 shows the model parameters used to fit the sorption isotherm at 23 °C for the different formulations. For GAB and Merakeb models, the E values are lower than 10 and the correlation coefficients for all models used are close to 1. Thus, GAB and Merakeb models are considered appropriate [45].

### 3.6. Moisture Buffer Value 

**Figure 15:**
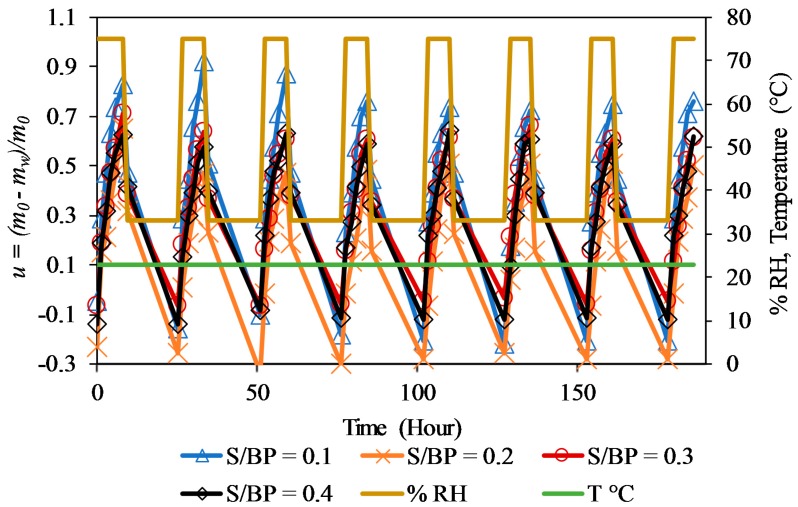
Moisture uptake and release of S-BP concrete during cyclic relative humidity variation in climatic chamber.

Figure 15 presents the moisture content *u* of S-BP composites during variations of relative humidity between 33% and 75% at 23 °C, where *m_w_* is the absorbed or released water mass and *m*_0_ is the initial mass of the sample. This figure also shows the ability of the composite to absorb moisture at 75% RH and to release moisture at 33% RH. For the last three cycles, the mass variation of the samples appears to have the stabilized condition.

The results presented in Table 6 clearly show that the composite can be classified as an excellent regulator of the relative humidity of the environment (MBV > 2 g·m^−2^ % RH^−1^) according to the classification proposed by Rode [42]. The samples present the MBV values between 2.6 and 2.8 g·m^−2^ % RH^−1^, which are comparable with the MBV values of hemp–starch composites [8].

It can be observed that the variation in MBV as a function of the S/BP mass ratio is linear and can be presented by Equation (12). Table 6 shows that increasing the mass of starch tends to increase the MBV value, because of the high moisture buffering of starch. The samples with more starch have the highest MBV value, around 2.80 g·m^−2^ % RH^−1^. These results apply to the uncoated S-BP composites and are expected to decrease when the coatings are used in order to enhance the durability of the composites.

(12) $$MBV=0.62\times \left(\frac{S}{BP}\right)+2.55$$

## 4. Conclusions

The hygrothermal and acoustical properties of S–BP composites were analyzed in this paper. Four formulations were studied with different S/BP mass ratios (0.1, 0.2, 0.3 and 0.4) to investigate the influence of the starch amount and the porosity on composite characterizations. The sound absorption coefficient of the composite varied depending on the humidity amount and the porosity. The better acoustical performance was obtained under ambient conditions (50% RH and 23 °C) and with a lower starch amount (S/BP = 0.1).

The thermal conductivity, thermal diffusivity, and effusivity results showed that the S-BP composite can be used as a good thermal insulator. This composite showed the same thermal conductivity of several insulating materials [46]. The increase of starch amount tends to increase the thermal conductivity of the composites.

The results showed that the water vapor permeability depends on the porosity and the sorption isotherm is independent of the S/BP mass ratio.

The MBV values increased linearly from 2.6 g·m^−2^ % RH^−1^ to 2.8 g·m^−2^ % RH^−1^ when the mass ratio S/BP increased from 0.1 to 0.4, thus indicating that the S-BP composite can be an excellent moisture regulator when it is uncoated.

## Figures and Tables

**Table 1:** Porosity and densities of BP.

Aggregates	*ρ*_app_ (kg·m^−3^)	*ρ*_abs_ (kg·m^−3^)	Porosity (%)
Fresh beet pulp	134 ± 6.7	911.6 ± 45.6	85.3
Extruded beet pulp	194 ± 7.9	1073.4 ± 53.7	81.9

**Table 2:** Thermal properties of S-BP composites at 23 °C.

S/BP Mass Ratio	*λ* (W·m^−1^·K^−1^)	*a* (m^2^·s^−1^)	*b* (J·K^−1^·m^−2^·s^−1/2^)
0.1	0.069 ± 0.0006	1.76 ± 0.058 × 10^−7^	165.5 ± 3.6
0.2	0.071 ± 0.0005	1.66 ± 0.0713 × 10^−7^	175.2 ± 3.8
0.3	0.072 ± 0.0003	1.50 ± 0.052 × 10^−7^	186.8 ± 3.5
0.4	0.075 ± 0.0002	1.47 ± 0.042 × 10^−7^	±5.7

**Table 3:** Thermal conductivities of apparent agro-materials *λ_exp_* and solid particles *λ_s_* at 23 °C according to the self-consistent scheme.

Samples	*λ_exp_* (W·m^−1^·K^−1^)	*n*	*λ_s_*
S/BP 0.1	0.069	0.7779	0.299
S/BP 0.2	0.071	0.7559	0.283
S/BP 0.3	0.072	0.7413	0.272
S/BP 0.4	0.075	0.7216	0.267
Beet pulp	0.062	0.8193	0.315
Starch gel	0.1396	0.3857	0.2411

**Table 4:** Permeability and resistance to water vapor of S-BP composites.

Mass Ratio (S/BP)	*δ_v_* × 10^−12^ (kg·m^−1^·s^−1^·Pa^−1^)	*μ*
0.1	8.90 ± 0.211	22.48 ± 0.54
0.2	7.77 ± 0.169	25.72 ± 0.55
0.3	7.35 ± 0.154	27.20 ± 0.58
0.4	6.86 ± 0.236	29.12 ± 1.01

**Table 5:** Parameter values for the sorption isotherm models.

Models	Parameters	Samples S/BP = 0.1
Merakeb	*a*	1.5425
	*b*	0.2410
	*u_s_*	0.2211
	*E (%)*	1.1689
	*R* ^2^	0.9998
	*RMSE*	0.1114
GAB	*W_m_*	2.2848
	*C_G_*	0.0938
	*K*	0.4024
	*E (%)*	5.5864
	*R* ^2^	0.9994
	*RMSE*	0.2484
Van Genuchten (VG)	*U_s_*	0.1921
	*η_T_*	2.2379
	*α_T_*	0.0002
	*E (%)*	10.4050
	*R* ^2^	0.9982
	*RMSE*	0.3936

*a, b:* intrinsic thermodynamic parameters for the Merakeb model, *u_s_*: saturation moisture content by mass, *C_G_* and *K*: dimensionless parameters for the GAB model related to heat of sorption in the monolayer and multilayer region, respectively, *η_T_* and *α_T_*: characteristic constants of VG model.

**Table 6:** Moisture buffer values of S-BP composites.

Sample S/BP	MBV (g·m^−2^ % RH^−1^)	SD
0.1	2.62	0.060
0.2	2.68	0.065
0.3	2.72	0.080
0.4	2.80	0.090
